# From Functional Disorder to a Life-Threatening Emergency: A Rare Adolescent Case of Thyrotoxicosis

**DOI:** 10.7759/cureus.101630

**Published:** 2026-01-15

**Authors:** Séphora Chanhoun, Hugo Picardo, Gauthier Leclercq, Laurent Pâques, Emma Carles, Fabien Guerisse

**Affiliations:** 1 Emergency Medicine, Centre Hospitalier Universitaire de Charleroi-Chimay, Charleroi, BEL; 2 Faculty of Medicine, Université de Mons, Mons, BEL

**Keywords:** adolescent, endocrine emergency, hyperthyroidism, thyroid storm, thyrotoxicosis

## Abstract

Thyroid storm is a rare but life-threatening endocrine emergency that requires rapid recognition and multidisciplinary care. We report the case of a 16-year-old female patient admitted to the emergency department with acute confusion, fever, and tachycardia, in whom severe thyrotoxicosis was confirmed. Her Burch-Wartofsky score was 40, consistent with an impending thyroid storm. Graves’ disease had been previously diagnosed, but treatment was delayed, and influenza B infection acted as a precipitating factor. Management with antithyroid drugs, beta-blockers, and intensive care led to clinical improvement, with thyroidectomy subsequently planned. This case highlights the importance of early diagnosis, coordinated management, and awareness of infection as a trigger, while emphasizing the limitations of antithyroid therapy in adolescents.

## Introduction

The endocrine system maintains homeostasis through hormonal regulation of essential body functions [1,2]. Key components include the hypothalamic-pituitary axis and specialized glands like the thyroid, parathyroids, adrenals, pancreas, and gonads. Endocrine disorders cause diverse metabolic imbalances, often chronic, but sometimes acute emergencies like diabetic complications, adrenal crises, or thyroid dysfunctions [3]. A French study noted that, though rare in emergency departments, endocrine disorders are linked to high morbidity, particularly with delayed diagnosis [4]. Hyperthyroidism and its severe form, thyroid storm, are rare but critical emergencies, with 10%-20% mortality, requiring swift diagnosis and multidisciplinary care [5-8]. We report a case of thyroid storm managed at CHU Charleroi-Chimay (Belgium), highlighting its diagnostic and therapeutic challenges.

## Case presentation

A 16-year-old female patient was transported to the hospital by the mobile emergency medical service and admitted to the emergency department for acute confusion, heart palpitations, and fever. Upon emergency evaluation, she was placed in a resuscitation bay with continuous monitoring; her medical history was limited due to altered consciousness, though her parents reported flu-like symptoms with a productive cough since the previous day. Clinical examination revealed BP 121/61 mmHg, HR 108 bpm, RR 26/min, and SpO₂ 98% in ambient air. She was agitated and pale, with regular heart sounds and symmetrical breath sounds, while the abdomen was diffusely tender without guarding. Neurologically, she presented with aphasia, a Glasgow Coma Scale score [9] of 11/15 (E3V2M6), bilaterally reactive miosis, mobilization of all limbs without lateralization, and no meningeal signs.

**Figure 1:**
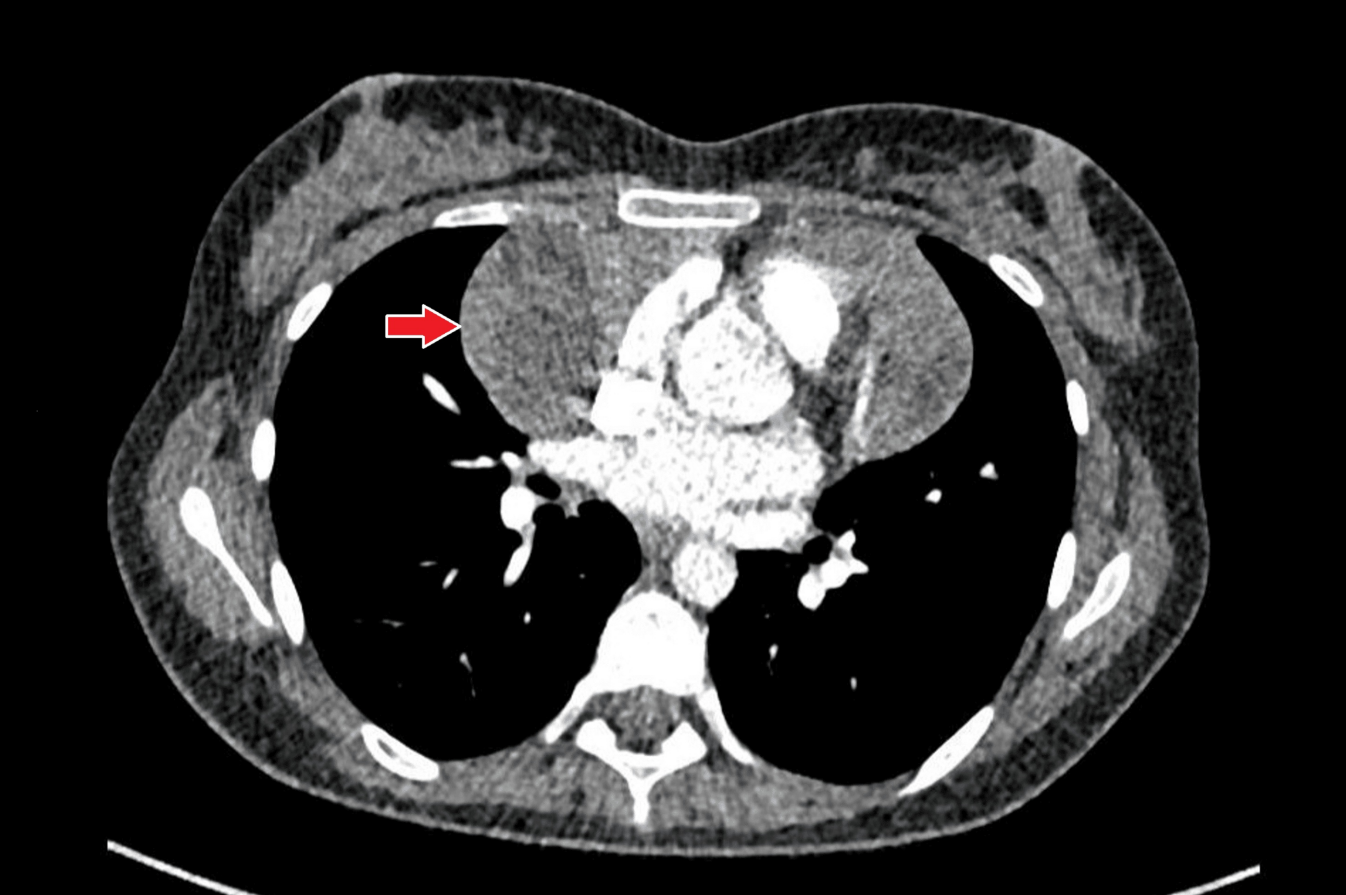
Chest CT angiography demonstrating an anterior mediastinal mass

**Figure 2:**
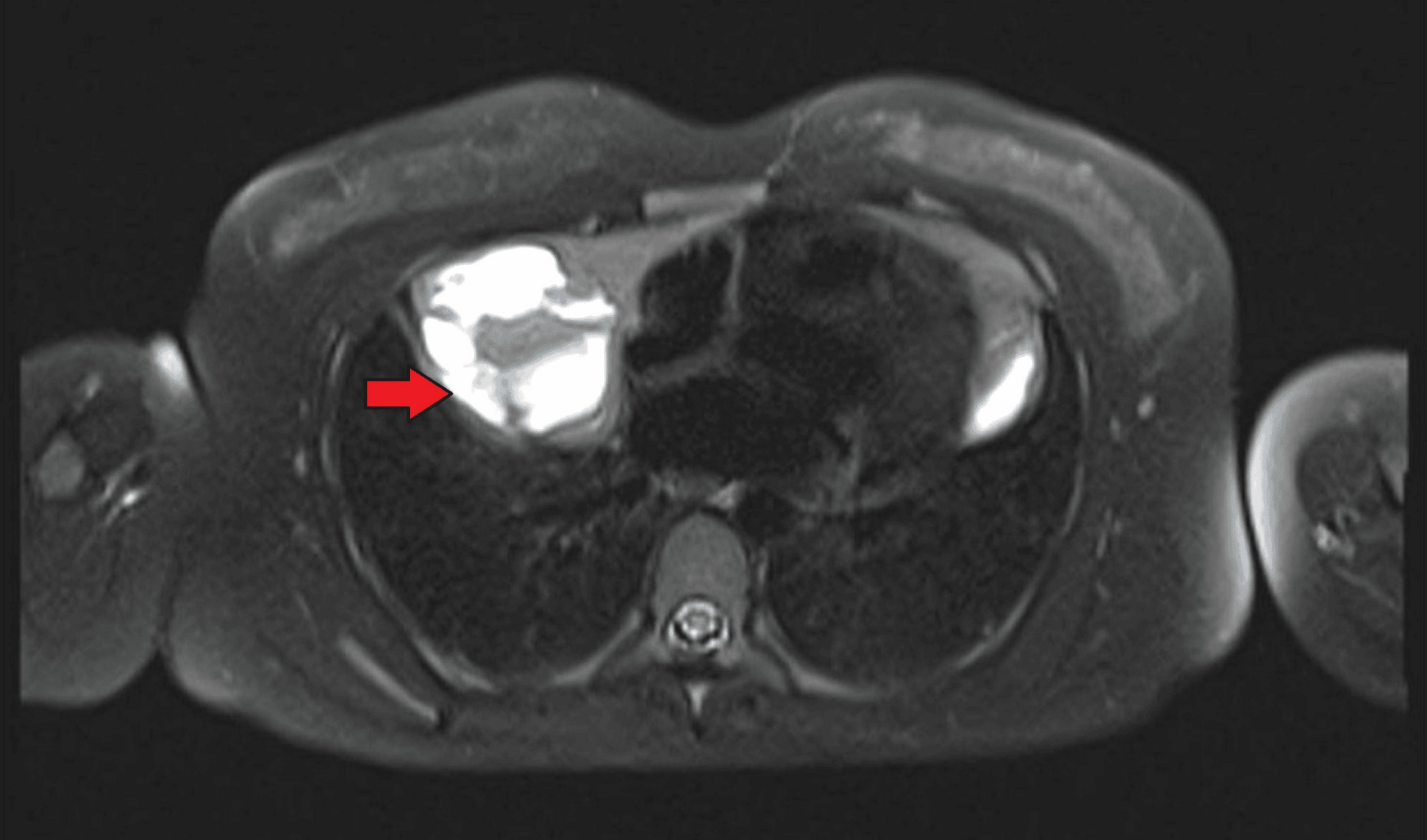
Whole-body PET scan demonstrating an anterior mediastinal mass without abnormal tracer uptake, compatible with a benign cystic lymphangioma

Medical history was significant for no addictions or allergies, though four months earlier, the patient had been admitted to the emergency department with abdominal pain, sinus tachycardia (122 bpm), and SpO₂ 94%. Laboratory tests at that time showed elevated D-dimers (1.40 µg/mL) and hyperthyroidism (TSH < 0.04 mUI/L (N 0.35-4.94), free T4 40.1 pmol/L (N 9-19)), while chest CT angiography (Figure 1) ruled out pulmonary embolism but revealed a mediastinal mass, characterized by subsequent PET scan (Figure 2) and MRI as a benign cystic lymphangioma. Endocrinology follow-up was delayed until one month prior to admission, when she reported palpitations, mood disturbances, and insomnia.

Her vital signs upon examination were BP 120/60 mmHg and HR 100 bpm, with lab tests showing TSH < 0.04 mU/L, free T4 33.0 pmol/L, free T3 > 30.72 pmol/L, and TSI 23.0 UI/L. Methimazole (Strumazol 10 mg, 2 tablets twice daily) was initiated, but two weeks later, lab tests showed persistent thyrotoxicosis (TSH < 0.04 mU/L, free T4 40.3 pmol/L, free T3 > 30.72 pmol/L, TSI 21.8 UI/L), and ultrasound imaging of the neck revealed a hypoechoic, heterogeneous gland with Doppler hyperemia consistent with thyroiditis.

**Figure 3:**
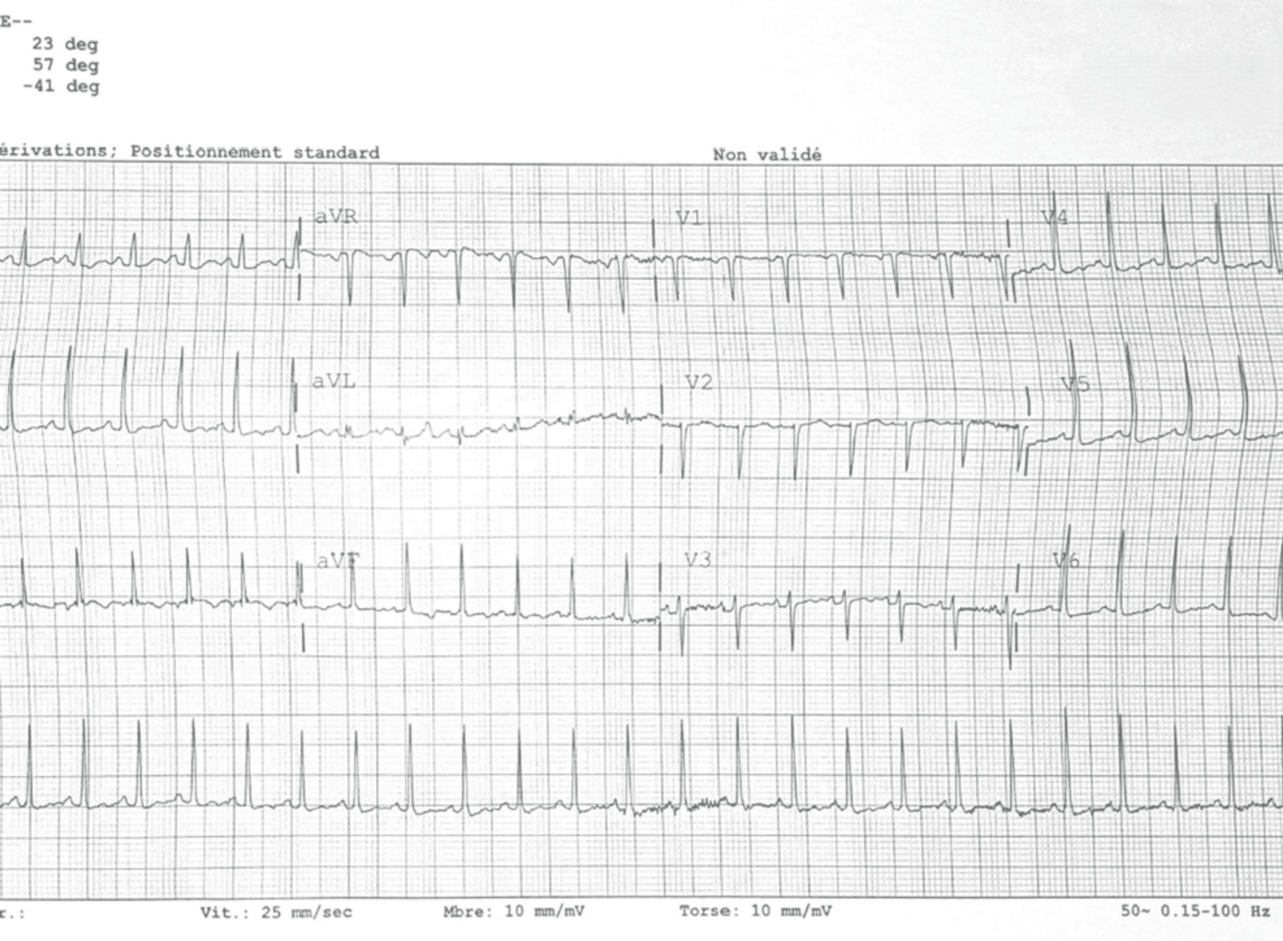
ECG performed upon admission in the resuscitation bay, confirming a regular sinus rhythm at 108 bpm, without repolarization abnormalities or signs of ventricular overload

Upon current admission to the emergency department, investigations revealed a positive nasopharyngeal swab for influenza B, while urine toxicology was negative. The ECG (Figure 3) showed sinus tachycardia at 108 bpm, with narrow QRS, no ST abnormalities, and no ventricular overload. Chest X-ray was normal. Brain CT excluded acute intracranial events. Lumbar puncture was performed, and cerebrospinal fluid analysis was negative. Laboratory results (Table 1) included Hb 12.5 g/dL, WBC 4200/mm³, CRP 1 mg/L, creatinine 0.46 mg/dL, bilirubin 0.31 mg/dL, TSH < 0.04 mU/L, free T4 > 60 pmol/L (undetectable), pH 7.44, pCO₂ 28 mmHg, pO₂ 100 mmHg, and lactates 0.6 mmol/L.

**Table 1:** Laboratory results on the day of admission to the emergency department

Variable	Result	Reference range
Hemoglobin (Hb)	12.5 g/dL	12-16 g/dL (women); 13-17 g/dL (men)
White blood cells (WBC)	4200/mm³	4000-11,000/mm³
C-reactive protein (CRP)	1 mg/L	<5 mg/L
Creatinine	0.46 mg/dL	0.52-0.94 mg/dL
Total bilirubin	0.31 mg/dL	0.10-0.84 mg/dL
Thyroid-stimulating hormone (TSH)	<0.04 mUI/L	0.4-4.0 mIU/L
Free T4 (FT4)	>60 pmol/L (undetectable)	12-22 pmol/L
pH	7.44	7.35-7.45
pCO₂	28 mmHg	35-45 mmHg
pO₂	100 mmHg	75-100 mmHg
Lactate	0.6 mmol/L	0.5-1.5 mmol/L

Regarding the initial management, two IV lines were placed, and a single dose of intravenous ceftriaxone 2 g was administered. Once the investigations confirmed the diagnosis of severe thyroid storm with altered consciousness, the patient was admitted to the ICU for 24 hours, where treatment included propylthiouracil (PTU) 50 mg (2 tablets twice daily) and propranolol (Inderal) 40 mg (1 tablet twice daily). During the 10-day hospital course in internal medicine, the patient presented low-grade fever, persistent cough, and developed agranulocytosis, induced by antithyroid drugs. At discharge, treatment included Inderal 40 mg twice daily, potassium iodide 65 mg twice daily, and Strumazol 10 mg (two tablets thrice daily), with surgery recommended once euthyroidism is achieved. Two months later, due to toxicity and resistance to Strumazol, treatment was switched to PTU 50 mg (two tablets twice daily, then thrice daily) and Inderal 40 mg thrice daily. Thyroidectomy was scheduled the following week, with postoperative labs confirming euthyroidism.

## Discussion

Hyperthyroidism and thyroid storm represent a continuum of disease. Thyroid storm results from the massive release of hormones, causing acute metabolic overstimulation [5-7,10].

Thyrotropin‑releasing hormone (thyrotropin) from the hypothalamus stimulates pituitary thyroid‑stimulating hormone (TSH), which drives thyroid secretion of triiodothyronine (T3) and thyroxine (T4). In hyperthyroidism, feedback control fails, and in thyroid storm, hormone levels rise to toxic concentrations [8,11-13].

Hyperthyroidism affects 1.2%-1.6% of the population and is often subclinical [8,14]. Graves’ disease is the leading cause in children and adolescents [15,16], followed by Hashimoto thyroiditis [17]. Pediatric incidence varies: 4.58/100,000 in France [18], lower in Europe (1.83 in Denmark, 2.7 in Sweden, 0.9 in the United Kingdom) [19], and higher in Hong Kong (5-6.5/100,000). A Turkish study found Graves’ disease in 74.6% of 503 pediatric cases [20]. In Belgium, no data is currently available.

Thyroid storm occurs in 2%-16% of hospitalized thyrotoxicosis patients, with mortality ranging from 1.2% to 10%, depending on the series [7,21-23].

Clinical features of hyperthyroidism include tachycardia, palpitations, irritability, tremor, weight loss, sweating, heat intolerance, sleep disturbance, asthenia, proximal weakness, menstrual irregularities, and goiter. Thyroid storm is an exacerbated form, with hyperthermia, agitation, confusion, coma, severe tachycardia, arrhythmia, multiorgan failure, and high mortality.

Ali et al. reported an atypical “apathetic” thyroid storm in a 38-year-old man with undiagnosed Graves’ disease, presenting with severe cardiopulmonary failure and multiorgan involvement but without agitation or fever [24]. Bonfield and Shenoy (2018) described a four-year-old girl with Graves’ disease who developed acute thyroid storm with tachycardia, hypertension, diffuse goiter, and exophthalmos, resolving within two weeks under carbimazole and propranolol [25].

Our patient presented with fever, sustained tachycardia, agitation, and confusion, without major hemodynamic collapse.

Thyroid storm may be precipitated by infections, surgery, trauma, delivery, abrupt withdrawal of antithyroid drugs, or medications (amiodarone, iodine) [13,24,26,27]. In this case, *Haemophilus influenzae *B infection and delayed treatment initiation were identified.

The initial triad of fever, tachycardia, and confusion is nonspecific, mimicking infection or neurological/metabolic disorders. In this context, lumbar puncture and empirical ceftriaxone were justified until results became available.

Biological diagnosis of hyperthyroidism relies on suppressed TSH (<0.04 mUI/L) and markedly elevated free T3 and free T4. Graves’ disease is confirmed by positive TRAb/TSI antibodies, often associated with anti-TPO or anti-Tg antibodies. Complementary examinations, such as blood pressure, heart rate, ECG, and thyroid ultrasound, contribute to clinical evaluation [17,20]. The Burch-Wartofsky Point Scale [28] (Table 2) is a useful tool to estimate the probability of thyroid storm by scoring clinical and biological signs [13,26,27]. In our patient, retrospective assessment yielded a score of 40, corresponding to moderate probability.

**Table 2:** Burch-Wartofsky Point Scale (BWPS) for assessing the probability of thyroid storm This scale has been made freely available under a Creative Commons (CC-BY-NC-ND) license: https://creativecommons.org/licenses/by-nc-nd/2.0/. Source: [28].

Category	Criteria	Value/manifestation	Points
Thermoregulation	Temperature (°F/°C)	99.0-99.9/37.2-37.7	5
		100.0-100.9/37.8-38.3	10
		101.0-101.9/38.4-38.8	15
		102.0-102.9/38.9-39.4	20
		103.0-103.9/39.5-39.9	25
		≥104.0/≥40	30
Cardiovascular	Tachycardia (bpm)	90-109	5
		110-119	10
		120-129	15
		130-139	20
		≥140	25
	Congestive heart failure	Absent	0
		Mild	5
		Moderate	10
		Severe	15
	Atrial fibrillation	Absent	0
		Present	10
Gastrohepatic	Symptoms	None	0
		Moderate (diarrhea, abdominal pain, nausea/vomiting)	10
		Severe (jaundice)	20
CNS	Symptoms	None	0
		Mild (agitation)	10
		Moderate (delirium, psychosis, extreme lethargy)	20
		Severe (seizure, coma)	30
Precipitating factor	Presence	Negative	0
		Positive	10
Score interpretation	≥45: thyroid storm		
	25-44: impending storm		
	<25: storm unlikely		

Without treatment, hyperthyroidism tends to worsen, and spontaneous remission is rare [17]. Current strategies rely on antithyroid drugs (methimazole or PTU) either with dose titration or block-and-replace protocols using levothyroxine. Definitive options include thyroidectomy or radioactive iodine (I-131), both of which induce permanent hypothyroidism requiring lifelong substitution [17,20]. Antithyroid drugs are first-line treatment, but relapses are frequent. PTU is not recommended in children due to the risk of severe hepatotoxicity, except in specific situations such as pregnancy [20,29]. In pregnant women, PTU is preferred in the first trimester, then replaced by methimazole to limit hepatic toxicity [22].

Adjunctive therapies include beta-blockers (propranolol), iodine (Lugol) administered after antithyroid drugs in preparation for surgery, corticosteroids to inhibit peripheral conversion of T4 to T3, and antipyretics (excluding aspirin). Severe refractory cases may require extracorporeal techniques. Lim et al. (2021) described a 28-year-old patient with thyroid storm complicated by cardiac arrest and multiorgan failure, treated with plasmapheresis, continuous dialysis, and extracorporeal membrane oxygenation (ECMO), but with a fatal outcome [26].

In our patient, initial therapy combined methimazole and propranolol. During thyroid storm, PTU was introduced in the ICU, then methimazole was resumed. Two months later, persistent thyrotoxicosis led to a switch back to PTU. These successive adjustments raise questions regarding adherence to current recommendations, which discourage prolonged PTU use. Earlier surgical intervention might have been considered.

Perspectives

Conventional treatments remain effective but limited by tolerance, relapse, and complications. Emerging targeted therapies, including cytokine inhibitors and monoclonal antibodies against the TSH receptor, may offer more personalized options [30]. Lim et al. (2021) reported extracorporeal therapies achieving 85% survival in 27 refractory cases [26]. A Japanese multicenter prospective study showed that implementation of national guidelines (Japan Thyroid Association, 2016) reduced 30-day mortality from 10.7% to 5.5%, despite higher severity scores (APACHE II median 13 vs 10). Adherence to protocols significantly lowered mortality in severe cases (APACHE II ≥12: 50% vs 4.7%, p = 0.01), supporting the integration of standardized guidelines into international practice [31].

## Conclusions

This case illustrates the diagnostic and therapeutic complexity of thyroid storm in adolescents and emphasizes the need for effective coordination across different levels of care. It serves as a valuable educational tool to raise awareness among clinicians about endocrine emergencies, the importance of rapid multidisciplinary management, and the need for a high index of suspicion. Furthermore, it highlights challenges related to delays in access to specialized care and the optimization of therapeutic strategies in pediatrics, opening perspectives for clinical and organizational research.
